# Role of Electrostatic Hotspots in the Selectivity of Complement Control Proteins Toward Human and Bovine Complement Inhibition

**DOI:** 10.3389/fmolb.2021.618068

**Published:** 2021-03-16

**Authors:** Yogesh B. Narkhede, Avneesh K. Gautam, Rohaine V. Hsu, Wilson Rodriguez, Nehemiah T. Zewde, Reed E. S. Harrison, Pablo R. Arantes, Zied Gaieb, Ronald D. Gorham, Chris Kieslich, Dimitrios Morikis, Arvind Sahu, Giulia Palermo

**Affiliations:** ^1^ Department of Bioengineering, University of California, Riverside, CA,; ^2^ National Centre for Cell Science, Pune University Campus, Ganeshkhind, India; ^3^ Department of Chemical Engineering, Auburn University, Auburn, AL,; ^4^ Department of Chemistry, University of California, Riverside, CA,

**Keywords:** Complement cofactor activity, Computational modeling, SPICE, VCP, Variola (smallpox) virus

## Abstract

Poxviruses are dangerous pathogens, which can cause fatal infection in unvaccinated individuals. The causative agent of smallpox in humans, *variola virus*, is closely related to the bovine *vaccinia virus*, yet the molecular basis of their selectivity is currently incompletely understood. Here, we examine the role of the electrostatics in the selectivity of the smallpox protein SPICE and vaccinia protein VCP toward the human and bovine complement protein C3b, a key component of the complement immune response. Electrostatic calculations, in-silico alanine-scan and electrostatic hotspot analysis, as introduced by Kieslich and Morikis (*PLoS Comput. Biol*. 2012), are used to assess the electrostatic complementarity and to identify sites resistant to local perturbation where the electrostatic potential is likely to be evolutionary conserved. The calculations suggest that the bovine C3b is electrostatically prone to selectively bind its VCP ligand. On the other hand, the human isoform of C3b exhibits a lower electrostatic complementarity toward its SPICE ligand. Yet, the human C3b displays a highly preserved electrostatic core, which suggests that this isoform could be less selective in binding different ligands like SPICE and the human Factor H. This is supported by experimental cofactor activity assays revealing that the human C3b is prone to bind both SPICE and Factor H, which exhibit diverse electrostatic properties. Additional investigations considering mutants of SPICE and VCP that revert their selectivity reveal an “electrostatic switch” into the central modules of the ligands, supporting the critical role of the electrostatics in the selectivity. Taken together, these evidences provide insights into the selectivity mechanism of the complement regulator proteins encoded by the *variola* and *vaccinia viruses* to circumvent the complement immunity and exert their pathogenic action. These fundamental aspects are valuable for the development of novel vaccines and therapeutic strategies.

## Introduction

Poxviruses are highly dangerous pathogens, whose most notable member is *variola virus*, the causative agent of smallpox, which killed millions of people before the massive vaccination campaign by the World Health Organization (World Health Organization, 1980). Closely related to *variola virus*, the vaccine-derived agent, called *vaccinia virus*, is causing outbreaks in dairy cattle in India and Brazil (Trindade et al., 2007; Gurav et al., 2011). Intriguingly, while these poxviruses are closely related, *variola virus* is strictly human-specific, whereas *vaccinia virus* also infects bovines. However, the molecular basis of this selectivity is currently incompletely understood. Its knowledge is fundamental to understand the onset of smallpox outbreaks dairy cattle, as arising from *vaccinia virus*, as well as to develop novel vaccines and therapeutic strategies.

**FIGURE 1:**
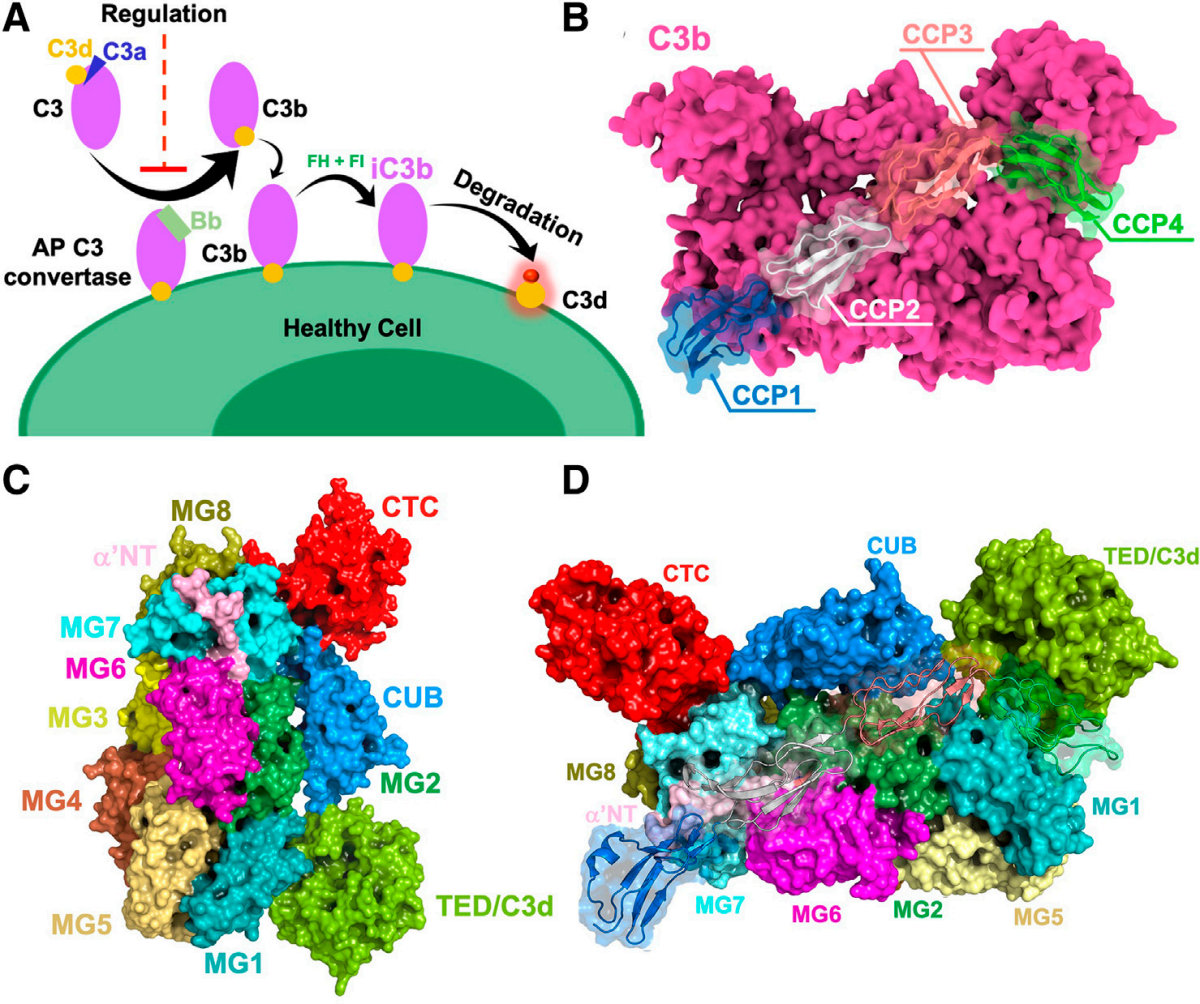
**(A)** General overview of complement activation and regulation on healthy cells. Once complement is activated, the active fragment C3b is generated by the C3 convertase. Further cleavage events yield inactivated iC3b, which gets converted to C3d (yellow). **(B)** Human complement C3b protein bound to the human Factor H (FH), as captured via x-ray crystallography (PDB ID: 2WII) (Wu et al., 2009). The C3b protein is depicted as a molecular surface (magenta), binding the first four complement control protein (CCP) modules of FH (CCP1-4, shown using different colors). **(C)** Surface representations of complement C3b domains shown in different colors. **(D)** Complement C3b in complex with CCP1-4 of human FH. Domains CUB, TED, MG1, MG2, MG6, MG7, and MG8 of C3b provide a binding interface for human FH.

Both viruses actively evade the complement system, which is an effector arm of the immune system and one of the major innate immune responses to viruses. Thanks to an intricate network of proteins, the complement system regulates important pathophysiological functions, eliminates pathogens, ensures homeostasis and forms a bridge between the innate and adaptive immunity (Bennett et al., 2017; Reis et al., 2019). When the complement system is activated, the convertase enzyme converts the complement 3 (C3) protein into its C3b component, which can associate to cell surfaces (Figure 1A). Further cleavage events yield C3d (the terminal cleavage product of C3), which is a natural biomarker of complement activation. Both *variola* and *vaccinia* viruses encode complement regulator proteins, named SPICE (smallpox inhibitor of complement enzymes) and VCP (vaccinia virus complement control protein) respectively, which are structural homologues of the proteins that regulate complement activation. Specifically, both SPICE and VCP mimic the interactions of the human Factor H (FH, which is structurally similar to SPICE and VCP), such as competing with FH in binding C3b (Figure 1B). At the domain level, human FH mainly interacts with the following domains: CUB, TED, MG1, MG2, MG6, MG7, and MG8 (Figures 1C,D; Supplementary Figure S1). There are two mechanisms of complement regulation in humans—cofactor activity (CA) and decay accelerating activity (DAA). The cofactor activity involves a cofactor (e.g., FH/VCP/SPICE) and a plasma serine protease factor I (FI) that proteolytically degrades the complement proteins C3b/C4b, while decay-accelerating activity involves the decay or dissociation of the catalytic domain of the convertases. With these mechanisms, the smallpox and vaccinia viruses circumvent the complement immunity, exerting their pathogenic function (Lambris et al., 2008; Agrawal et al., 2017; Rosbjerg et al., 2017). At the molecular level, the complement regulator proteins SPICE, VCP, and the human FH consist of bead-like repeating subunits, known as Complement Control Protein (CCP) modules, which are covalently linked forming “beads-on-a-string” structures (Ojha et al., 2014). SPICE, VCP, and bovine FH display a sequence identity with the human FH of 27, 33, and 65%, respectively (Supplementary Figure S2) (Altschul et al., 1990; Gish and States, 1993). However, the first four CCP modules that bind C3b display a similar architecture and a sequence identity >90% with the human FH (Figure 1B; Supplementary Figure S2) (Wu et al., 2009). Indeed, only the K108 and K120 of SPICE correspond to E108 and E120 in VCP, which suggests a change in the electrostatic properties (Yadav et al., 2012). Considering the similarity of SPICE and VCP, it is intriguing how these two viral proteins could achieve selectivity toward the human and bovine isoforms of C3b (Yadav et al., 2012). This has been further highlighted by experimental studies that show the SPICE-HuC3b interaction has a K_D_ of 1.3 μM (Forneris et al., 2016), while that of Hu FH(1-4)-HuC3b interaction is 11.0 μM (Wu et al., 2009). When relative binding was measured using SPR, the relative binding strength of SPICE for HuC3b was ∼130-fold higher than for VCP (Yadav et al., 2008), and relative binding strength of VCP for BoC3b was ∼2.6 fold higher than for SPICE (Yadav et al., 2012). Several computational studies have revealed a key role of the electrostatics in the binding of CCP modules to the complement receptors, (Sfyroera et al., 2005; Zhang and Morikis, 2006; Yadav et al., 2008; Liszewski et al., 2009; El-Assaad et al., 2011; Kieslich et al., 2011c; Kieslich and Morikis, 2012; Yadav et al., 2012; Harrison et al., 2015; Mohan et al., 2015; Harrison et al., 2020), demonstrating that the diverse electrostatic properties of CCP modules mediate different specificities toward the complement proteins. Kieslich and Morikis (Kieslich and Morikis, 2012) have examined the effect of electrostatic interactions at the level of the complement C3 fragment C3d and its complement receptor 2 (CR2), whose formation constitutes the link between innate and adaptive immunity, proposing that the C3d-CR2 electrostatic specificity is critical in the onset of adaptive immunity. That study demonstrated that functional “electrostatic hotspots” importantly contribute to the protein-protein association and to the selectivity of C3d and its receptor. In this respect, electrostatic hotspots comprising clusters of charged residues have been found to be crucial regulators also across several other biomolecular systems, functioning also as effective drug binding sites (Foloppe et al., 2006; Adhireksan et al., 2017).

In this work, we examine the role of the electrostatics in the selectivity of SPICE and VCP toward the human and bovine isoforms of C3b. Electrostatic calculations, and in-silico alanine-scanning of SPICE, VCP, and human factor H bound to human and bovine C3b have been performed to characterize electrostatic hotspots, as introduced by Kieslich and Morikis (Kieslich and Morikis, 2012), identifying sites resistant to local perturbation where the electrostatic potential is likely to be evolutionary conserved (Kieslich and Morikis, 2012). The calculations suggest that the bovine C3b is electrostatically prone to selectively bind its VCP exogenous ligand. On the other hand, the human isoform of C3b exhibits a lower electrostatic complementarity toward its SPICE ligand. Yet, the human C3b displays a highly preserved electrostatic core, which suggests that this isoform could be less selective and bind different ligands, such as both SPICE and the human FH. This is supported by experimental Cofactor Activity Assays revealing that the human C3b is prone to bind both SPICE and FH, which exhibit a diverse ESI map and different electrostatic properties. Further investigations of the SPICE and VCP mutants reverting their selectivity (Yadav et al., 2012) reveal that an “electrostatic switch” into their central modules plays a key role in the process. Overall, these evidences highlight the critical role of the electrostatics in the specificity of SPICE and VCP toward the human and bovine isoforms of the C3b protein, providing fundamental insights that can help the development of novel vaccines and therapeutic strategies.

## Computational Materials and Methods

### Structure Preparation

Structural models have been based on the crystallographic coordinates of the structure of the human C3b-FH complex (PDB ID: 2WII, Figure 1B) (Wu et al., 2009) solved at 2.7 Å resolution. SPICE and VCP display a sequence identity (Madeira et al., 2019) with the endogenous human FH of 27 and 33% respectively (Altschul et al., 1990; Gish and States, 1993). However, our investigations focused on the first four CCP modules that bind C3b, which display a similar architecture and a sequence identity >90% with the human FH (Wu et al., 2009). The Bovine C3b, SPICE, and VCP structures were modeled using PDB 2WII as a template through homology modeling using the software Modeller (Eswar et al., 2006). The human C3b portion of 2WII served as a reference for bovine C3b, while factor H was used to model the structures of VCP and SPICE. Following the homology modeling, an iterative qualitative assessment (Williams et al., 2018) and refinement of the models was performed using UCLA SAVES server (SAVES, 2020) and Modeller. The final models were aligned to PDB 2WII yielding the following complexes: Human C3b-SPICE, Human C3b-VCP, Bovine C3b-SPICE, and Bovine C3b-VCP.

### Computational Alanine-Scan, Electrostatic Calculations, and Hotspot Analysis

Electrostatic hotspot analysis has been performed using the AESOP (Analysis of Electrostatic Similarities Of Proteins) code, developed by Morikis (Kieslich et al., 2011b; Harrison et al., 2017) and extensively employed for electrostatic analysis of proteins (Kieslich et al., 2011a, Kieslich et al., 2011b; Gorham et al., 2011a, Gorham et al., 2011b; Harrison et al., 2015; Mohan et al., 2015, Mohan et al., 2016; Zewde et al., 2018; Harrison and Morikis, 2019). AESOP enables the analysis of electrostatic hotspots through the calculation of an Electrostatic Similarity Indices (ESI), (Kieslich and Morikis, 2012), which assesses the electrostatic potential upon perturbation (i.e., mutagenesis), identifying regions of high electrostatic similarity, or those regions least affected by perturbation. High ESI values identify regions with resistance to perturbation, where the electrostatic potential is likely to be evolutionarily conserved, and constitute functional sites or electrostatic hotspots (Kieslich and Morikis, 2012). The calculation of the ESI involves the following steps: 1) generation of alanine scan mutations for every ionizable residue in a protein complex, one at a time, 2) calculation of Poisson-Boltzmann electrostatic potentials for each mutant protein and parent, 3) calculation of ESI values and projection on the protein surface for visualization. The resulting surface maps can be referred to as ESI perturbation maps, displaying regions of the protein resistant to perturbation (i.e., displaying high ESI values) and identifying electrostatic hotspots. Upon alanine-scan and calculation of the electrostatic potentials, cumulative distribution of Electrostatic Similarity Index (ESI) is computed as:

$$\text{ESI}\left(i,j,k\right)=\frac{1}{N}\sum_{n=1}^{N}1-\frac{\left|\varphi_{A}\left(i,j,k\right)-\;\varphi_{B,n}\left(i,j,k\right)\right|}{\max\left(\left|\varphi_{A}\left(i,j,k\right)\right|,\left|\varphi_{B,n}\left(i,j,k\right)\right|\right)},$$

where $\varphi_{A}$ is the electrostatic potential of the original protein and $\varphi_{B,n}$ is the electrostatic potential of the N mutants at the grid point (i,j,k). The ESI is calculated at each grid point (i,j,k) and normalized by the number of electrostatic potential comparisons N. This measure of electrostatic similarity describes the similarity of the electrostatic potential of a set of mutants to the original parent protein at a given grid point, for a family of *N* mutants. For the ESI calculations, an ionic strength corresponding to 0 mM monovalent ion concentration was used, as described earlier (Kieslich and Morikis, 2012). As described above, electrostatic hotspots can be evaluated as the projections of the ESI values on the protein surface.

In this work, the number of protein mutants generated by alanine scan are as follows: 66 mutants for FH, 42 mutants for SPICE, 46 mutants for VCP, 394 mutants for the human C3b, and 419 mutants for the bovine C3b. The calculations were performed at pH 7, mutating the following ionizable amino acids: Asp, Glu, Arg, Lys, and His. The apparent pK_a_ of the His residues was pre-determined using PROPKA (Søndergaard et al., 2011). Electrostatic potentials were calculated by solving the linearized Poisson-Boltzmann equation using APBS (Baker et al., 2001). Positional coordinates in the PDB files were converted to PQR files in preparation for the Poisson-Boltzmann calculations, by adding hydrogen atoms, atomic radii, and partial charges, using PDB2PQR (Dolinsky et al., 2007) and the PARSE force field (Sitkoff et al., 1994). Electrostatic calculations were performed for the families of mutant and parent original proteins, using the separated protein and ligand components from the protein-ligand complex. The protein-ligand complex structure was centered in a grid with 161 × 161 × 257 grid points and grid lengths of 144 × 156 × 228 Å, resulting in a resolution of less than 1 Å/grid point. The protein or ligand was then removed to performed electrostatic potential calculations of each component. All mutants of each protein were centered in an identical manner to assure proper comparison of electrostatic potentials. Ionic strength corresponding to concentration of monovalent solvent ions of 150 mM was used in the calculations. Protein and solvent dielectric coefficients were set to 20 and 78.54, respectively, as determined in previous studies (Gorham et al., 2011b). Calculations were performed at 298.15 K temperature. Additional electrostatic potential analysis was performed for the VCP mutants E108K, E120K, and E108K/E120K, and the SPICE mutants K108E, K120E, K108E/K120E/N144E. Electrostatic calculations have been performed as described above, using ionic strengths corresponding to 150 mM. The software Chimera (Pettersen et al., 2004) has been employed to project ESI values on the 3D structures, thereby visualizing the electrostatic hotspots.

### Cofactor Activity Assay

The cofactor activity of factor H in conjunction with factor I toward human and bovine C3b was performed by employing a fluid phase cofactor assay described earlier (Gautam et al., 2015). In brief, 2 μg of C3b (human or bovine) was mixed with 1.1 μg of factor H in presence or absence of 15 ng of factor I (human or bovine) in a total volume of 15 μl, and reaction mix was incubated at 37 °C for different time intervals. After the indicated time points, 5 μl of the sample buffer containing DTT was added to the reaction mix to stop the reaction. The C3b cleavage products were then resolved on 9% SDS-PAGE gel. The percentage of C3b cleaved with time was quantitated by densitometric analysis of α′-chain of C3b. The time required for 50% cleavage of the α′-chain of C3b was calculated from the plots generated by plotting the percentage of α′-chain of C3b against time. Human and bovine C3b, and bovine factor I were purified from the respective plasma as described (Yadav et al., 2012). Human factor H and factor I were purchased from Complement Technology, Inc. (TX, United States). Although it would be more meaningful to test bovine endogenous ligand (i.e., bovine FH) to test our hypothesis, however, our efforts to purify bovine FH from the sera failed, and the same is not available commercially.

## Results and Discussion

The results of homology modeling for the bovine complement C3b, SPICE, and VCP have been summarized in Supplementary Table S1 and Supplementary Figures S3–S6. The analyses revealed that the models are of sufficient quality for the ensuing computational electrostatic calculations.

**FIGURE 2:**
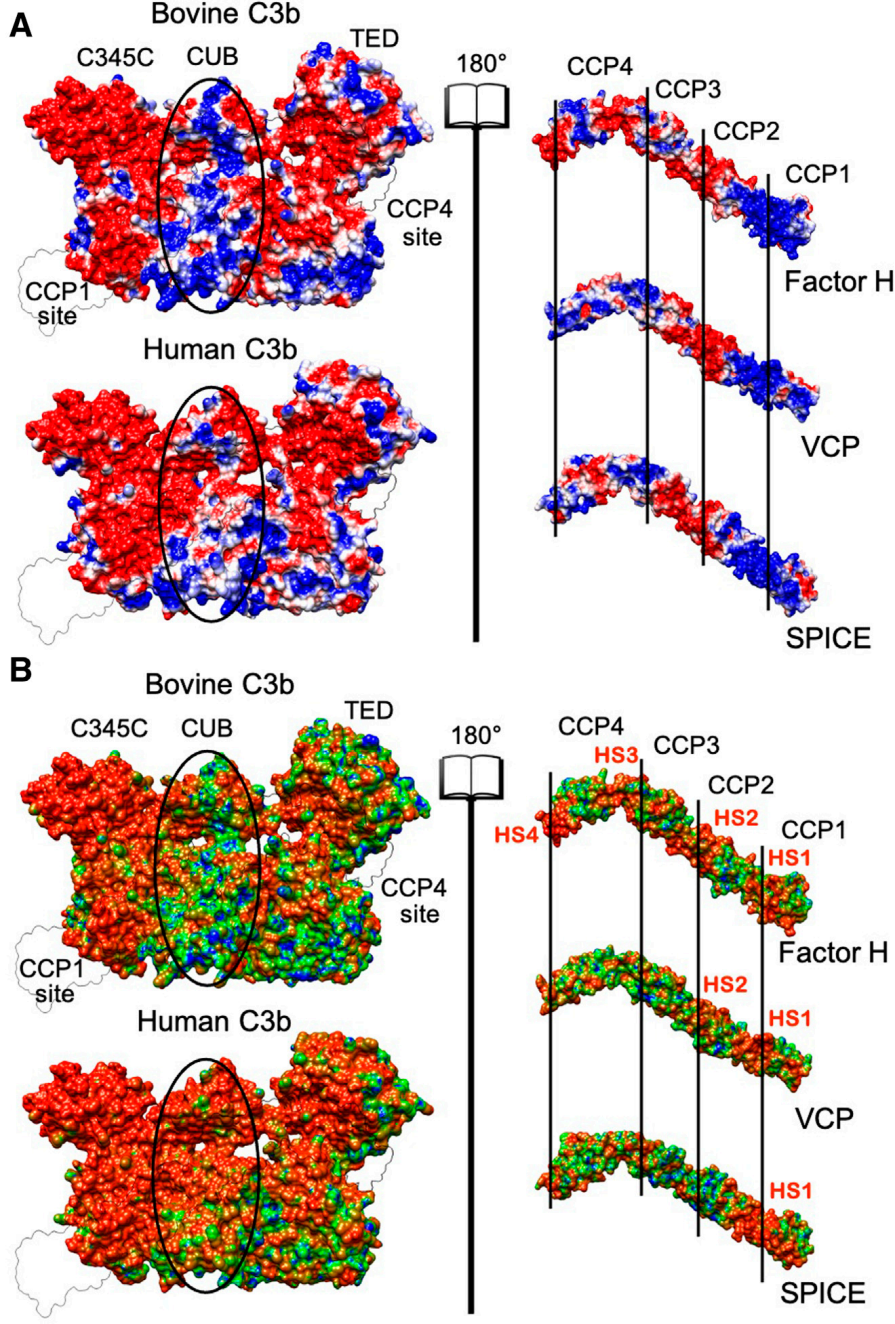
**(A)** Electrostatic potential surface projection for the bovine and human C3b proteins (left panel) and for the Factor H, VCP and SPICE ligands (right panel). The left and right panels are separated by an “open book” view, which reveals the surfaces of contact at the interface of the protein-ligand complex. The protein domains of C3b (C345C, CUB, and TED) and the complement control protein (CCP) modules of Factor H, VCP and SPICE (CCP1-4) are also shown. On the 3D structure of C3b, the location of the CCP1 and CCP4 binding sites is also shown. The ellipses depict differences in the central regions of bovine and human C3b. The electrostatic potential is colored from red (negative) to blue (positive). **(B)** Electrostatic Similarity Index (ESI), projected on the 3D structure of the bovine and human C3b protein (left panel) and of the Factor H, VCP and SPICE ligands (right panel). Observed electrostatic hotspots on the ligands are marked as HS1–HS4. The color scheme blue–green–orange represents low–to–high similarity, corresponding to ESI values of 0.6–0.8–1.0 for the bovine and human C3b, and ESI values of 0.4–0.7–1.0 for the ligands.

In order to understand the role of the electrostatics in the complementarity between C3b and its ligands, we performed electrostatic calculations, thereby analyzing the spatial distributions of the electrostatic potential. Figure 2A shows the surface projections of the electrostatic potentials for the bovine and human C3b, as well as for its ligands FH, VCP, and SPICE. An “open book” view highlights the surfaces of contact at the interface of the protein-ligand complex. The surface projections of the electrostatic potential of both bovine and human C3b are overall similar. However, a more negative character in the CUB domain of the human C3b was observed, compared to the bovine protein (net charges are –30e^−^ for human C3b and –16e^−^ for bovine C3b). The surface projections of the electrostatic potential of the ligands, FH, VCP, and SPICE, display both similarities and pronounced differences. Electrostatic complementarity between both the bovine and human C3b proteins and their ligands is evident at the level of the positively charged ligand module CCP1. Indeed, the CCP1 module of FH, VCP, and SPICE exhibits electrostatic complementarity with the negatively charged MG7 domain, where the CCP1 module binds (Figure 2A) (Wu et al., 2009). Additionally, our hotspot analyses reveal that the CUB and MG6 domains of bovine and human C3b harbor most of the variations that play a key role in CCP2 binding for FH, SPICE, and VCP (Figure 2B). Partial electrostatic complementarity is observed between module CCP4 of FH, VCP, and SPICE and the TED domain of both bovine and human C3b, although the distribution of positive and negative patches in CCP4 differs in FH, VCP, and SPICE. In the case of the middle modules CCP2 and CCP3 of VCP and SPICE (which display a more negative electrostatic surface), the electrostatic complementarity appears to be favored with the bovine C3b, with respect to the human C3b, which shows increased negatively charged surface. This observation agrees with the increased specificity observed for VCP toward the bovine C3b (Yadav et al., 2012). However, it does not explain the lower specificity of binding for SPICE, which is instead human complement-specific.

As discussed before, (Kieslich and Morikis, 2012) visual inspection of the electrostatic potential may not adequately highlight the electrostatic contributions to protein-protein association. Hence, to gain further insights into the electrostatic complementarity, we employed an Electrostatic Similarity Index (ESI) (Carbó et al., 1980; Hodgkin and Richards, 1987). This analysis enables to assess the electrostatic potential upon perturbation (i.e., mutagenesis, details are reported in the Methods section), identifying regions of high “electrostatic similarity” or those regions least affected by perturbation. High ESI values indicate regions with resistance to perturbation with evolutionarily conserved electrostatic potential (Kieslich and Morikis, 2012), and pinpoints the presence of electrostatic hotspots. Regions of C3b and its ligands characterized by high ESI values are shown in Figure 2B, as identified by the surface projection of ESI values on the 3D structures. The human C3b displays a larger electrostatic hotspot (orange surface) in the binding site of all ligand modules (CCP1-CCP4), extending to the C345C and CUB domains as well, compared to bovine C3b. A larger electrostatic hotspot is also observed in the TED domain of the human C3b, which is the binding site of the CCP4 module. The FH, VCP and SPICE ligands display however electrostatic hotspot diversity. An electrostatic hotspot in between modules CCP1-CCP2 is observed in all three ligands, but is more pronounced in SPICE (indicated in Figure 2B as Hot Spot 1, HS1). VCP and FH display a second hotspot at the CCP2-CCP3 interface (HS2), while FH also displays other two regions characterized by electrostatic hotspots at the level of CCP4 (HS3, HS4). Interpretation of the ESI maps suggests that HS1, which is conserved in the three ligands, mediates binding affinities for all three ligands at the level of the CCP1 binding site on both the human and bovine isoforms of C3b. A comparison with Figure 2A also shows that HS1 corresponds to positively charged areas, which are prone to bind the negatively charged binding site on C3b.

The bovine C3b, which preferentially binds VCP, shows that several electrostatic hotspots, where the electrostatics is resistant to perturbation, are distributed on the protein surface (Figure 2B). This is also observed in the VCP and FH ligands, where the HS1-3 also display a high electrostatic complementarity with the bovine C3b (Figure 2A). This is in line with the higher affinity of VCP for the bovine C3b, compared to human C3b (Yadav et al., 2012). Indeed, the negative charge of HS2 in VCP participates in attractive interactions with the positive charges in the CUB region of the bovine C3b, while establishing repulsive interactions with the negative charges in the CUB region of the human C3b (Figure 2A), thus contributing to the higher selectivity of VCP for bovine C3b.

The human C3b (which is SPICE-specific) shows an extended hotspot area, while SPICE shows the absence of hotspots in its complementary central modules CCP2 and CCP3 (Figure 2B). This observation indicates that while the human C3b strongly preserves its core electrostatics, its SPICE ligand is highly susceptible to electrostatic perturbation. Moreover, the central modules of SPICE display low electrostatic complementarity with the CUB region of the human C3b (Figure 2A) and the positively charged HS1 hotspot is the sole electrostatic anchor to the human C3b. The highly preserved electrostatic core indicated by the extended hotspot area in the human C3b suggests that this protein could be less selective in binding to different ligands like SPICE and FH, which exhibit diverse ESI map and different electrostatic properties (Yadav et al., 2012).

**FIGURE 3:**
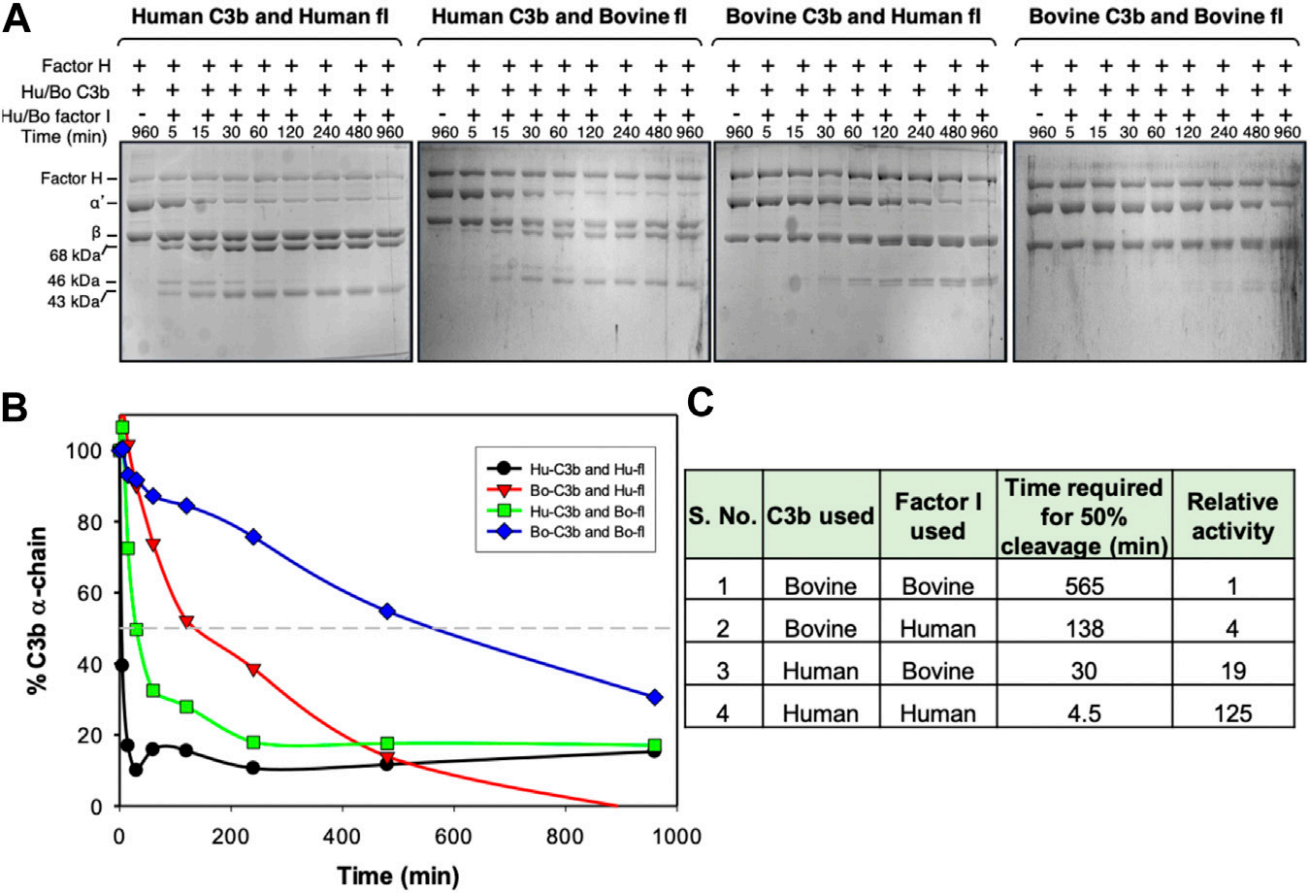
Cofactor activity of human factor H for human and bovine C3b. **(A)** Analysis of cofactor activity by SDS-PAGE gel analysis. C3b (human or bovine) was mixed with human factor H and factor I (human or bovine), incubated at 37 °C for the indicated time, and examined for C3b cleavage (α′-chain cleavage) by running the reaction mix on SDS-PAGE. **(B)** The amount of α′-chain at different time points was quantitated densitometrically and plotted against time. **(C)** Relative cofactor activity of factor H against human and bovine C3b in the presence of human or bovine factor I. The molar concentration of the complement components used in the cofactor assay was: 758 nM Hu/Bo C3b, 11 nM Hu/Bo factor I and 473 nM Hu factor H.

Overall, analysis of the ESI maps suggests that changes in the electrostatic properties of the central modules of VCP and SPICE (i.e., in between CCP2-CCP3) could modulate the selectivity for the bovine vs. the human C3b (Yadav et al., 2012). Most notably, the bovine C3b and its VCP ligand display a common heterogeneity of the ESI maps (Figure 2B) and a high electrostatic complementarity (Figure 2A). This suggests that the bovine C3b is electrostatically prone to bind its VCP and FH ligands, which exhibit similar electrostatic properties. The human isoform of C3b displays a large electrostatic hotspot, which indicates a highly preserved electrostatic core. On the other hand, its SPICE ligand shows the absence of hotspots in the central modules and low electrostatic complementarity, such that the positively charged HS1 hotspot is the sole electrostatic anchor to the human C3b. These evidences suggest that the human C3b, which exhibits a highly preserved electrostatic core, could be less selective and bind different ligands like SPICE and FH, which exhibit diverse ESI map and different electrostatic properties. To support this hypothesis, we performed cofactor activity assays, where the activity of the human FH at the level of the human C3b was measured (FH + HuC3b), in comparison with that of the human FH at the level of the bovine C3b (FH + BoC3b). These assays were performed in the presence of a critical serine protease enabling the reaction, *viz.* factor I, both from bovine and human species (Figure 3). Compared to our controls (FH + HuC3b + HuFI), a 31-fold decrease in cofactor activity was observed when the bovine C3b is bound to the human FH (FH + BoC3b + HuFI), revealing that the bovine C3b is less prone to bind the human FH. Additionally, when comparing Cofactor activity Assay of FH + HuC3b + BoFI, and FH + BoC3b + BoFI, we observe a 19-fold decrease in relative activity, further supporting the evidence that the bovine C3b is less specific than the human C3b for the human FH. These cofactor activity assays were performed analogously to our previous experimental investigation describing the specificity of VCP and SPICE toward the bovine and human C3b, respectively (Yadav et al., 2012). Taken together, these experimental data indicate that the human C3b is prone to bind to the human FH (Figure 3), as well as its SPICE ligand (Yadav et al., 2012). Considering the remarkable differences in terms of electrostatic properties of the human FH and SPICE ligands (Figure 2), as well as the highly preserved electrostatic core of the human C3b, the above reported experimental evidence support that the human C3b is intrinsically less selective than the bovine C3b. Finally, it is notable that the experimental results reported in this paper, as well as the data from our previous study, have been performed by including the enzyme Factor I in the experimental assay. The binding of the FI enzyme occurs at a distal site with respect to the FH binding site on C3b, thereby not interfering with the FH-C3b interaction (Xue et al., 2017).

**FIGURE 4:**
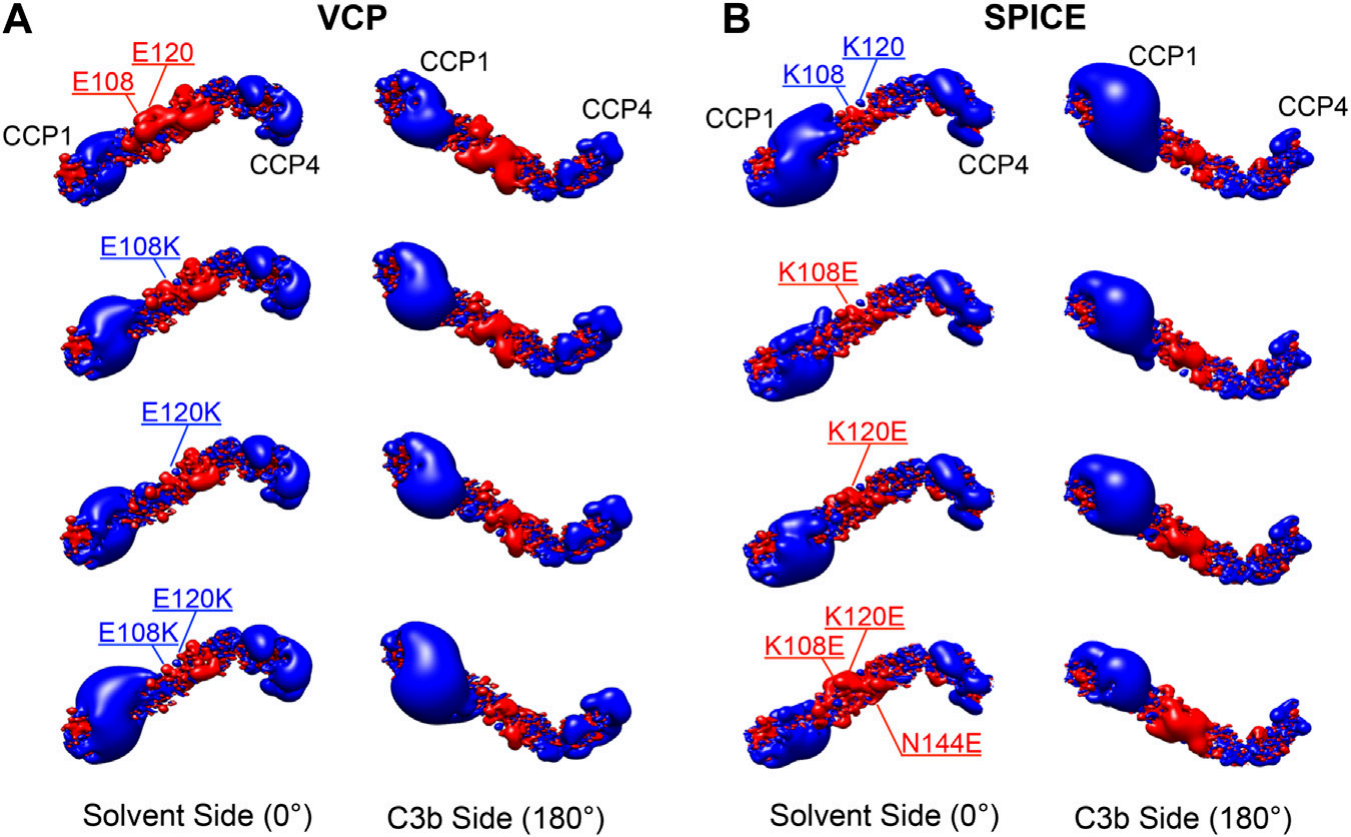
Electrostatic potential maps for the **(A)** Vaccinia virus complement control protein (VCP) and **(B)** smallpox inhibitor of complement enzymes (SPICE), upon point mutations of the E108K, E120K, and E108K/E120K residues in VCP, and upon the reverse mutations of K108E, K120E, and K108E/K120E/N144E in SPICE. The electrostatic potential is represented as isopotential contour maps, with blue and red contours having isovalues of +2 and −2 k_B_T/e^−^, respectively. Two orientations of the ligands are shown, displaying their solvent side and the side binding C3b (upon a 180° rotation around the horizontal axis). The electrostatic potential calculations were performed with 150 mM ionic strength.

**FIGURE 5:**
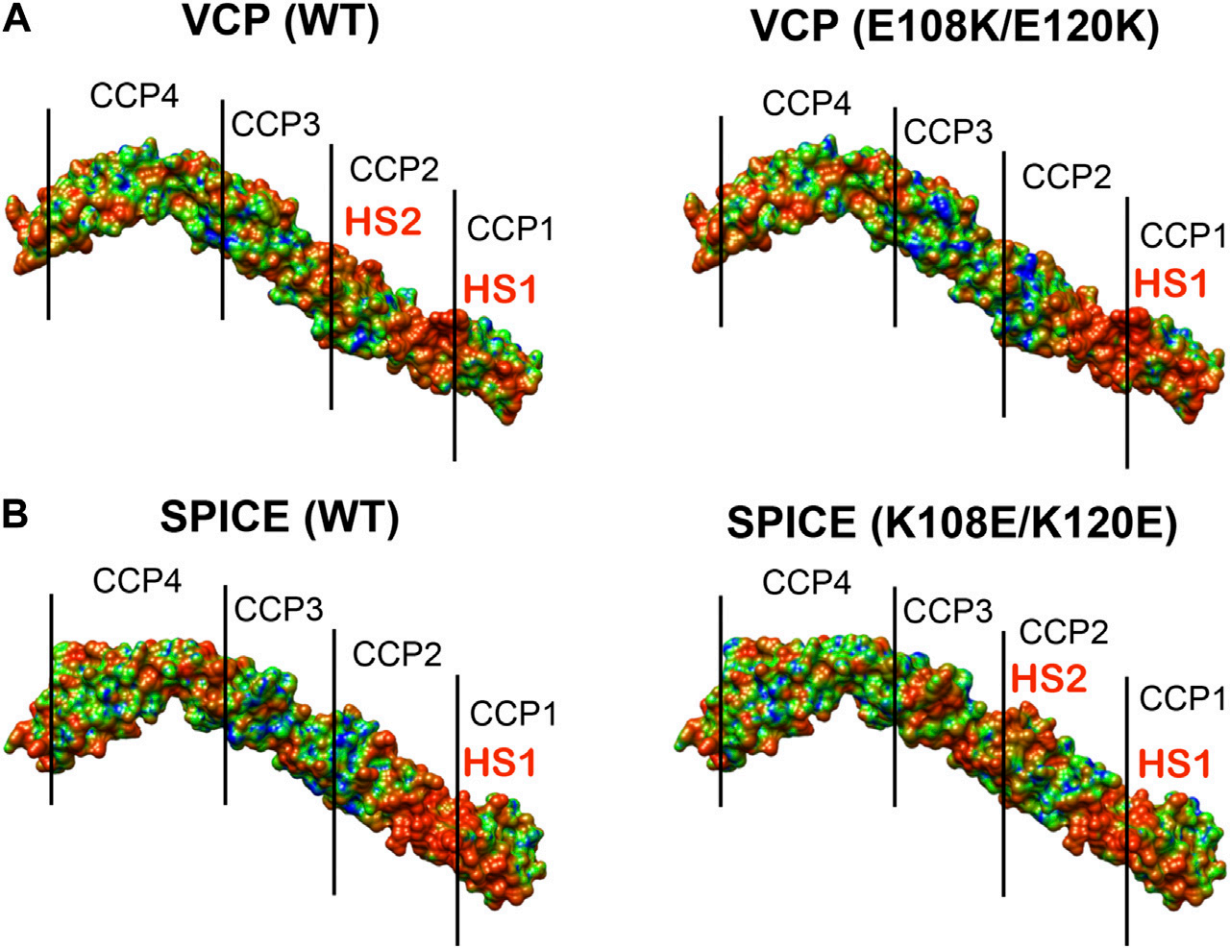
Electrostatic Similarity Index (ESI), projected on the 3D structure of **(A)** the vaccinia virus complement control protein (VCP) and of **(B)** the smallpox inhibitor of complement enzymes (SPICE). ESI is computed and projected on the proteins as wild-type (WT, left panel) and upon mutation of the residues 108 and 120 (right panel). Observed electrostatic hotspots on the ligands are marked as HS1–HS2. The color scheme blue–green–orange represents low–to–high similarity, corresponding to ESI values of 0.4–0.7–1.0.

To further investigate the electrostatic properties of SPICE and VCP, we extended our electrostatic analysis to two mutants of SPICE and VCP. We introduced mutations that have been experimentally shown to revert the selectivity toward the bovine and human C3b (Yadav et al., 2012). In SPICE, we introduced the K108E, K120E, and N144E mutations that confer bovine selectivity, whereas in VCP, we introduced the E108K, and E120K mutations that confer SPICE-like activity. Figure 4 shows the spatial distributions of electrostatic potentials of VCP (panel A), including the E108K, E120K, and E108K/E120K mutations, and of SPICE (panel B) including the reverse mutations (K108E ad K120E) and the N144E mutation, at an ionic strength of 150 mM. In VCP, the E108K and E120K mutations augment the positive electrostatic potential of modules CCP1 and CCP2. On the other hand, in SPICE, the K108E, K120E, and N144E mutations reduce the corresponding positive electrostatic potential. The increase of the positive electrostatic potential in the VCP modules CCP1 and CCP2, upon switching of the 108 and 120 residues from E–to–K, agrees well with a switch in selectivity toward the human C3b, which displays a highly negative electrostatic surface (Figure 2A) (Yadav et al., 2012). As opposite, the decrease of the positive electrostatic potential upon the reverse K–to–E mutations of residues 108 and 120 in SPICE, confers reduced affinity for the highly negatively charged electrostatic surface of the human C3b. Considering also the experimental evidence that these mutations revert the selectivity of the SPICE and VCP viral proteins, (Yadav et al., 2012) these outcomes suggest that an “electrostatic switch” into their central modules is critical for the opposite selectivity toward the human and bovine C3b. To gain more insights on this “electrostatic switch,” we performed the inspection of the electrostatic hotspots in the VCP and SPICE mutants through the calculation of the ESI maps. We analyzed the charge reversal for residues 108 and 120 in module CCP2 from E–to–K in VCP and from K–to–E in SPICE. This analysis has been carried out on the VCP E108K/E120K and SPICE K108E/K120E mutants, and compared with the wild-type ligands (Figure 5). As a result, the E108K/E120K mutation produces a VCP protein with a SPICE-like electrostatic hotspot map. Indeed, the E108K/E120K mutation augments the HS1 in VCP (to become similar to that of SPICE) and abolishes the HS2 hotspot (not present in SPICE). This is in good agreement with the experimental evidence that the E108K, E120K mutations in VCP confer to the latter SPICE-like activity (Yadav et al., 2012). On the other hand, the SPICE double mutation K108E/K120E generates a VCP-like electrostatic hotspot map, with reduction of the HS1 area and appearance of HS2. This confirms the evidence of an “electrostatic switch” in the central modules of SPICE and VCP.

## Conclusion

Here, we performed a computational study aimed at shedding light into the molecular mimicry of the viral proteins VCP and SPICE of the human FH binding to the complement protein C3b. These viral proteins exploit their structural similarity with the human FH to evade the immune response of the complement system, which is one of the important factors in their pathogenicity. Although SPICE and VCP are structural homologues and differ for a few amino acid mutations, they exhibit different selectivity toward the human and bovine isoforms of C3b (Yadav et al., 2012). Inspired by an early study by Kieslich and Morikis (Kieslich and Morikis, 2012), demonstrating that the diverse electrostatic properties of the Complement Control Proteins (CCP) mediate different specificities toward the complement proteins, we performed an electrostatic analysis of the SPICE and VCP ligands and of their human and bovine C3b counterparts. Electrostatic calculations, in-silico alanine-scan and electrostatic hotspot analysis were used to characterize the electrostatic complementarity of the protein-protein interaction and the onset of electrostatic hotspots, which are crucial regions where the electrostatic potential is resistant to permutation and is likely to be evolutionarily conserved (Kieslich and Morikis, 2012).

The calculations reveal that the bovine C3b, which preferentially binds VCP (Yadav et al., 2012), shows high electrostatic complementarity with its VCP ligand, displaying also several electrostatic hotspots that are also observed in VCP and FH. This suggests that the bovine C3b is electrostatically prone to selectively bind its VCP ligand. The human isoform of C3b displays a highly preserved electrostatic core, as arising from the observation of an extended electrostatic hotspot. On the other hand, its SPICE ligand shows the absence of hotspots in the central modules and low electrostatic complementarity, such that the positively charged HS1 hotspot is the sole electrostatic anchor to the human C3b. Considering this low electrostatic complementarity, the highly preserved electrostatic core in the human C3b suggests that this protein could be less selective in binding ligands like SPICE and FH. Additional investigations considering mutants of SPICE and VCP that revert their selectivity toward the bovine and human C3b (Yadav et al., 2012), reveal that an “electrostatic switch” into the central modules of the ligands is critical for their opposite selectivity. Taken together, these evidences highlight the critical role of the electrostatics in the specificity of SPICE and VCP toward the human and bovine isoforms of the C3b protein. Considering the pathogenesis of the *vaccinia virus*, causing outbreaks in dairy cattle in India and Brazil (Trindade et al., 2007; Gurav et al., 2011), the mechanistic aspects arising from this electrostatic analysis could provide critical insights to develop novel vaccines and therapeutic strategies.

## Data Availability

The raw data supporting the conclusion of this article will be made available by the authors, without undue reservation, upon request.
