# Supplementary material for: Role of Electrostatic Hotspots in the Selectivity of Complement Control Proteins Toward Human and Bovine Complement Inhibition
Source: Front Mol Biosci. 2021 Mar 16;8:618068. doi: 10.3389/fmolb.2021.618068 (PMC8020814; doi:10.3389/fmolb.2021.618068)
Supplement: Supplementary file 1 [file table1.docx]

Supporting Information

Role of Electrostatic Hotspots in the Selectivity of Complement Control Proteins toward Human and Bovine Complement Inhibition

Yogesh B. Narkhede,^1‡^ Avneesh K. Gautam^3‡^, Rohaine V. Hsu,^1‡^ Wilson Rodriguez,^1‡^ Nehemiah T. Zewde,^1^ Reed E. S. Harrison,^1^ Pablo R. Arantes,^1^ Zied Gaieb,^1^ Ronald D. Gorham Jr.,^1^ Chris Kieslich,^4^ Dimitrios Morikis,^1^ Arvind Sahu^5*^ and Giulia Palermo^1,2*^

**Table of Contents**

Supplementary Figures (S1-S6) …………………………………………………………..p. S2-S5

Supplementary Tables (S1) ……………………………………..............................................p. S6

Supplementary References (S1-47) ………………………………………………………p. S7-S10

Dedicated to our late beloved mentor Prof. Dimitrios Morikis.

Corresponding authors:

Giulia Palermo ([giulia.palermo@ucr.edu](mailto:giulia.palermo@ucr.edu))

Arvind Sahu ([arvindsahu@nccs.res.in](mailto:arvindsahu@nccs.res.in))

**Supplementary Figures**


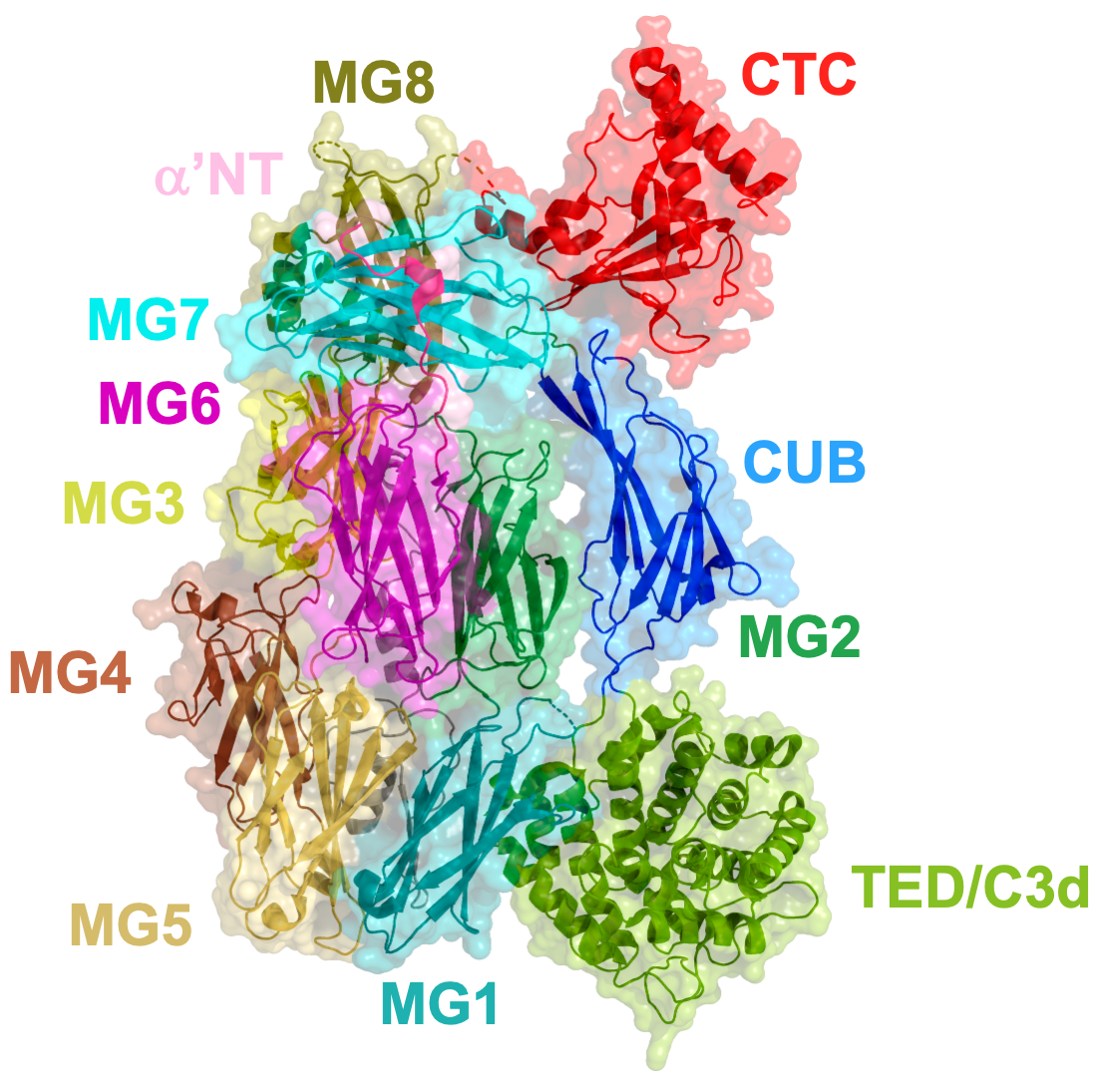


**Figure S1:** Ribbon and surface representations of complement C3b domains shown in different colors.


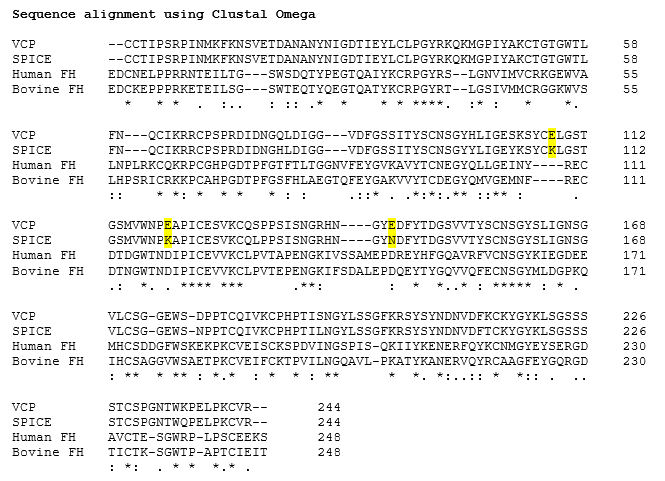


**Figure S2:** Multiple sequence alignment of VCP, SPICE, human factor H, and bovine factor H. The mutated positions have been highlighted in yellow.^47^


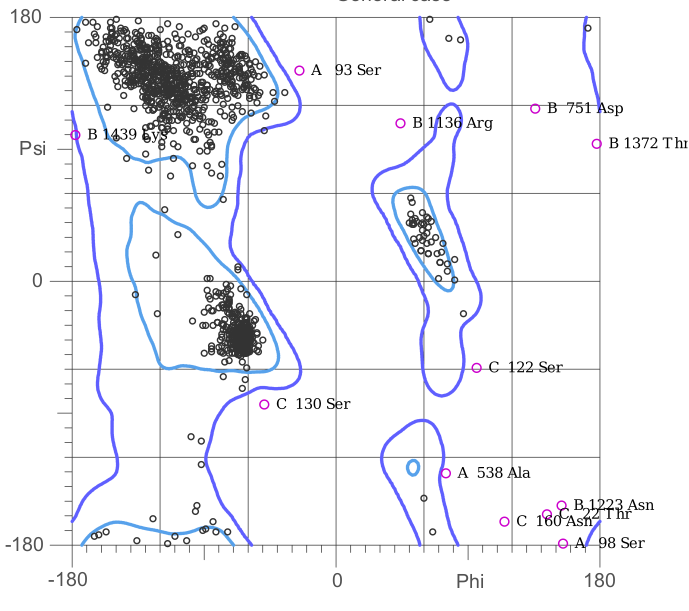


**Figure S3:** Ramachandran plot of Bovine C3b-VCP (wild type).


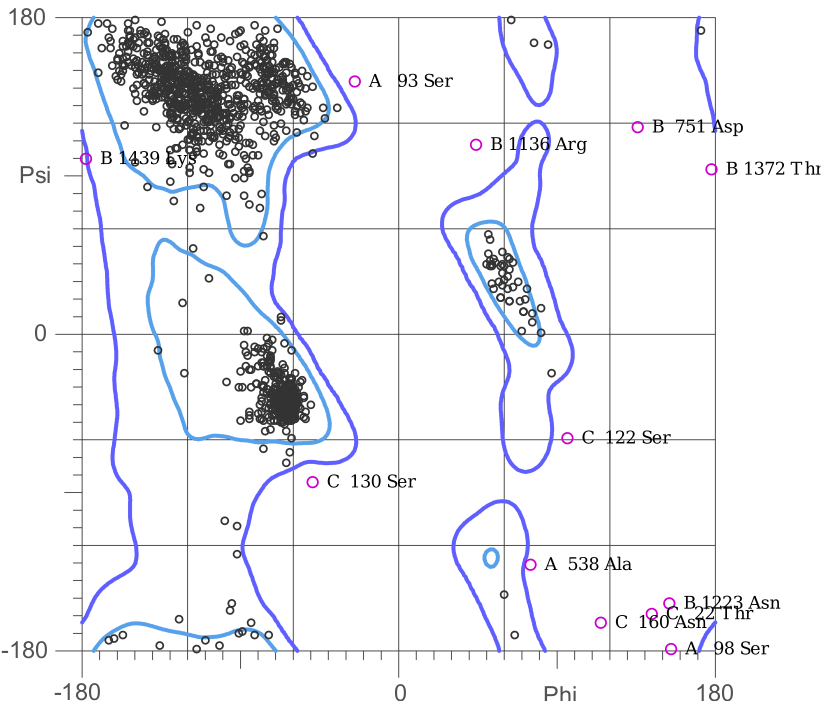


**Figure S4:** Ramachandran plot of Bovine C3b-VCP (mutant)


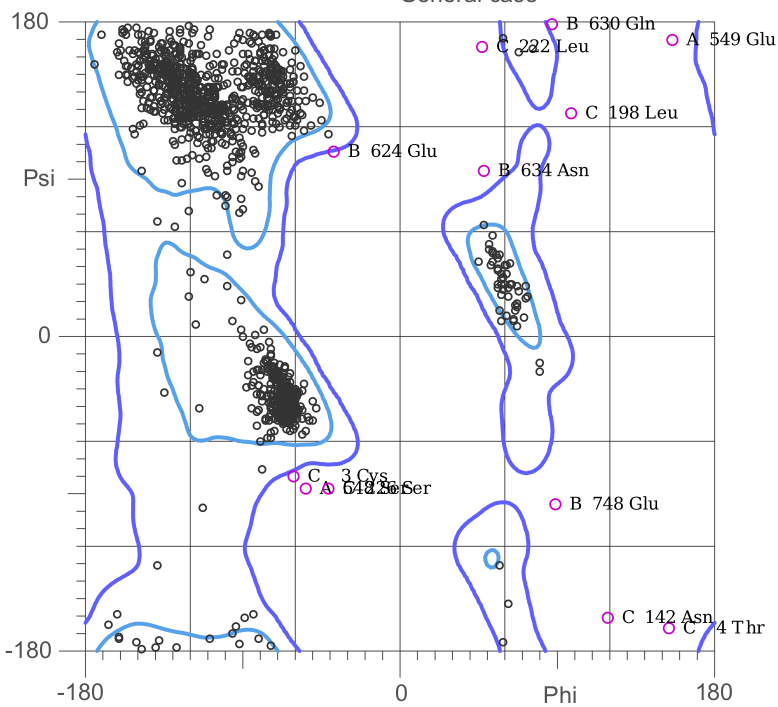


**Figure S5:** Ramachandran plot of Human C3b-SPICE (wild type)


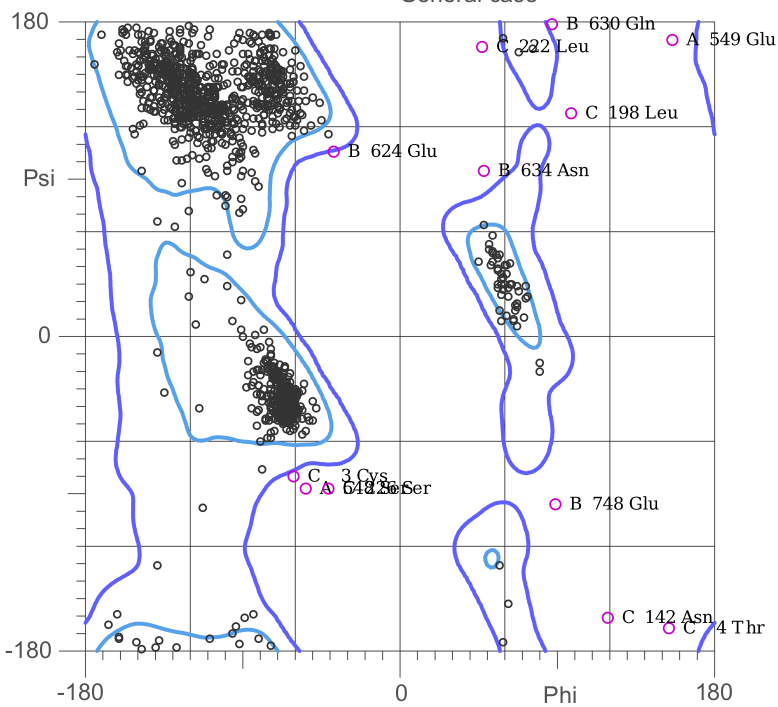


**Figure S6:** Ramachandran plot of Human C3b-SPICE (mutant)

**Supplementary Tables**

**Table S1:** Analysis of homology modelling for complexes of wild type and mutants of SPICE and VCP along with bovine C3b^28,46^

| Complex | ERRAT | Verify3D (%) | Ramachandran (% allowed) | Ramachandran Z-score |
| --- | --- | --- | --- | --- |
| Bovine C3b-VCP WT | 65.5968 | 85.91 | 96.61 | 0.07 ± 0.18 |
| Bovine C3b-VCP Mutant | 65.6725 | 85.91 | 96.71 | 0.07 ± 0.13 |
| Human C3b-SPICE WT | 76.5037 | 89.42 | 97.11% | 0.33 ± 0.18 |
| Human C3b-SPICE Mutant | 76.6161 | 89.47 | 97.10% | 0.32 ± 0.18 |

**Supplementary References**

1 W. H. Organization, *Wkly. Epidemiol. Rec.*, 1980, **55**, 145–152.

2 Y. K. Gurav, C. G. Raut, P. D. Yadav, B. V Tandale, A. Sivaram, M. D. Pore, A. Basu, D. T. Mourya and A. C. Mishra, *Prev. Vet. Med.*, 2011, **100**, 242–247.

3 G. S. Trindade, G. L. Emerson, D. S. Carroll, E. G. Kroon and I. K. Damon, *Emerg. Infect. Dis.*, 2007, **13**, 965–972.

4 E. S. Reis, D. C. Mastellos, G. Hajishengallis and J. D. Lambris, *Nat. Rev. Immunol.*, 2019, 19, 503–516.

5 K. M. Bennett, S. H. M. Rooijakkers and R. D. Gorham, *Front. Microbiol.*, 2017, 8.

6 P. Agrawal, R. Nawadkar, H. Ojha, J. Kumar and A. Sahu, *Front. Microbiol.*, 2017, 8.

7 A. Rosbjerg, N. Genster, K. Pilely and P. Garred, *Front. Microbiol.*, 2017, **8**, 868.

8 J. D. Lambris, D. Ricklin and B. V Geisbrecht, *Nat. Rev. Microbiol.*, 2008, **6**, 132.

9 H. Ojha, H. S. Panwar, R. D. Gorham, D. Morikis and A. Sahu, *Mol. Immunol.*, 2014, **61**, 89–99.

10 S. F. Altschul, W. Gish, W. Miller, E. W. Myers and D. J. Lipman, *J. Mol. Biol.*, 1990, **215**, 403–410.

11 W. Gish and D. J. States, *Nat. Genet.*, 1993, **3**, 266–272.

12 J. Wu, Y.-Q. Wu, D. Ricklin, B. J. C. Janssen, J. D. Lambris and P. Gros, *Nat. Immunol.*, 2009, **10**, 728–733.

13 V. N. Yadav, K. Pyaram, M. Ahmad and A. Sahu, *J. Immunol.*, 2012, **189**, 1431–1439.

14 F. Forneris, J. Wu, X. Xue, D. Ricklin, Z. Lin, G. Sfyroera, A. Tzekou, E. Volokhina, J. C. Granneman, R. Hauhart, P. Bertram, M. K. Liszewski, J. P. Atkinson, J. D. Lambris and P. Gros, *EMBO J.*, 2016, **35**, 1133–1149.

15 V. N. Yadav, K. Pyaram, J. Mullick and A. Sahu, *J. Virol.*, 2008, **82**, 3283–3294.

16 C. A. Kieslich and D. Morikis, *PLoS Comput. Biol.*, 2012, **8**, e1002840.

17 R. E. S. Harrison, N. T. Zewde, Y. B. Narkhede, R. V. Hsu, D. Morikis, V. I. Vullev and G. Palermo, *ACS Med. Chem. Lett*, 2020, **11**, 1054–1059.

18 R. R. Mohan, R. D. Gorham and D. Morikis, *Mol. Immunol.*, 2015, **64**, 112–122.

19 C. A. Kieslich, H. Vazquez, G. N. Goodman, A. L. de Victoria and D. Morikis, *J. Mol. Graph. Model.*, 2011, **29**, 1047–1055.

20 R. E. S. Harrison, R. D. Gorham and D. Morikis, *Protein Sci.*, 2015, **24**, 789–802.

21 A. M. El-Assaad, C. A. Kieslich, R. D. Gorham and D. Morikis, *Mol. Immunol.*, 2011, **48**, 1844–1850.

22 G. Sfyroera, M. Katragadda, D. Morikis, S. N. Isaacs and J. D. Lambris, *J. Immunol.*, 2005, **174**, 2143–2151.

23 L. Zhang and D. Morikis, *Biophys. J.*, 2006, **90**, 3106–3119.

24 M. K. Liszewski, M. K. Leung, R. Hauhart, C. J. Fang, P. Bertram and J. P. Atkinson, *J. Immunol.*, 2009, **183**, 3150–3159.

25 Z. Adhireksan, G. Palermo, T. Riedel, Z. Ma, R. Muhammad, U. Rothlisberger, P. J. Dyson and C. A. Davey, *Nat. Commun.*, 2017, **8**, 1–11.

26 N. Foloppe, N. Matassova and F. Aboul-ela, *Drug Discov. Today*, 2006, 11, 1019–1027.

27 N. Eswar, B. Webb, M. A. Marti‐Renom, M. S. Madhusudhan, D. Eramian, M. Shen, U. Pieper and A. Sali, *Curr. Protoc. Bioinforma.*, 2006, **15**, 5.6.1-5.6.30.

28 SAVES v5.0 - DOE-MBI Structure Lab UCLA, https://servicesn.mbi.ucla.edu/SAVES/, (accessed 22 December 2020).

29 C. A. Kieslich, D. Morikis, J. Yang and D. Gunopulos, *Biotechnol. Prog.*, 2011, **27**, 316–325.

30 R. E. S. Harrison, R. R. Mohan, R. D. Gorham, C. A. Kieslich and D. Morikis, *Biophys. J.*, 2017, **112**, 1761–1766.

31 R. D. Gorham, C. A. Kieslich and D. Morikis, *Ann. Biomed. Eng.*, 2011, **39**, 1252–1263.

32 C. A. Kieslich, R. D. Gorham and D. Morikis, *J. Non. Cryst. Solids*, 2011, **357**, 707–716.

33 R. D. Gorham, C. A. Kieslich, A. Nichols, N. U. Sausman, M. Foronda and D. Morikis, *Biopolymers*, 2011, **95**, 746–754.

34 R. E. S. Harrison and D. Morikis, *Biophys. J.*, 2019, **116**, 215–226.

35 N. Zewde, R. R. Mohan and D. Morikis, *Front. Phys.*, 2018, **6**, 130.

36 R. R. Mohan, G. A. Huber and D. Morikis, *J. Phys. Chem. B*, 2016, **120**, 8416–8423.

37 C. R. Søndergaard, M. H. M. Olsson, M. Rostkowski and J. H. Jensen, *J. Chem. Theory Comput.*, 2011, **7**, 2284–2295.

38 N. A. Baker, D. Sept, S. Joseph, M. J. Holst and J. A. McCammon, *Proc. Natl. Acad. Sci. U. S. A.*, 2001, **98**, 10037–10041.

39 T. J. Dolinsky, P. Czodrowski, H. Li, J. E. Nielsen, J. H. Jensen, G. Klebe and N. A. Baker, *Nucleic Acids Res.*, 2007, **35**, W522–W525.

40 D. Sitkoff, K. A. Sharp and B. Honig, *J. Phys. Chem.*, 1994, **98**, 1978–1988.

41 E. F. Pettersen, T. D. Goddard, C. C. Huang, G. S. Couch, D. M. Greenblatt, E. C. Meng and T. E. Ferrin, *J. Comput. Chem.*, 2004, **25**, 1605–1612.

42 A. K. Gautam, Y. Panse, P. Ghosh, M. J. Reza, J. Mullick and A. Sahu, *Proc. Natl. Acad. Sci. U. S. A.*, 2015, **112**, 12794–12799.

43 R. Carbó, L. Leyda and M. Arnau, *Int. J. Quantum Chem.*, 1980, **17**, 1185–1189.

44 E. E. Hodgkin and W. G. Richards, *Int. J. Quantum Chem.*, 1987, **32**, 105–110.

45 X. Xue, J. Wu, D. Ricklin, F. Forneris, P. Di Crescenzio, C. Q. Schmidt, J. Granneman, T. H. Sharp, J. D. Lambris and P. Gros, *Nat. Struct. Mol. Biol.*, 2017, **24**, 643–651.

46 C. J. Williams, J. J. Headd, N. W. Moriarty, M. G. Prisant, L. L. Videau, L. N. Deis, V. Verma, D. A. Keedy, B. J. Hintze, V. B. Chen, S. Jain, S. M. Lewis, W. B. Arendall, J. Snoeyink, P. D. Adams, S. C. Lovell, J. S. Richardson and D. C. Richardson, *Protein Sci.*, 2018, **27**, 293–315.

47 F. Madeira, Y. M. Park, J. Lee, N. Buso, T. Gur, N. Madhusoodanan, P. Basutkar, A. R. N. Tivey, S. C. Potter, R. D. Finn and R. Lopez, *Nucleic Acids Res.*, 2019, **47**, W636–W641.
